# A world of opportunity: A top-down influence of emotional intelligence-related contextual factors on employee engagement and exhaustion

**DOI:** 10.3389/fpsyg.2022.980339

**Published:** 2022-09-26

**Authors:** Zehavit Levitats, Zorana Ivcevic, Marc Brackett

**Affiliations:** ^1^Department of Political Studies, Bar-Ilan University, Ramat Gan, Israel; ^2^Yale Center for Emotional Intelligence, Yale University, New Haven, CT, United States

**Keywords:** organizational culture, emotional intelligence (EI), values, HRM practices, employee engagement, exhaustion, job demands-resources (JDR) model

## Abstract

Despite continuing interest in the impact of employees’ emotional intelligence (EI) in explaining for their engagement and emotional exhaustion, there are still large gaps in our understanding of the role played by contextual EI-related factors, such as an EI-related organizational culture and supervisors’ emotionally intelligent behavior (EIB). This two-study research approaches EI from a macro-level perspective, attempting to address three objectives: (1) to develop and define a theoretical concept of EI-supportive organizational culture, (2) to develop and validate measures of organizations’ EI-related values and practices, and (3) to investigate their top-down effect on employee engagement and exhaustion, *via* supervisor EI-related behavior. In the first study, we conceptualize and develop measures of perceived EI-related organizational values and human resource management (HRM) practices, as separate yet related dimensions of organizations’ EI-related culture, and test their validity. In the second study, we build on the job demands-resources (JD-R) theory and Ability-Motivation-Opportunity (AMO) framework to develop and test a model of the process links between perceived EI-related values and HRM practices and employee engagement and exhaustion, using a large sample of employees across industries in the USA workforce (*N* = 12,375). In line with our hypotheses, the findings suggest that EI-supportive HRM practices have a top-down effect on employee engagement and exhaustion *via* supervisor EIB, whereas low regard for emotions values has a top-down effect on employee exhaustion *via* supervisor emotional misbehavior. Results are discussed in the context of the JD-R theory, AMO framework, and the EI literature.

## Introduction

Much research on employee engagement and emotional exhaustion in workplace settings has focused on individual-level emotional antecedents of these outcomes (e.g., Brackett et al., 2010; Miao et al., 2017; Extremera et al., 2018). Employee emotional intelligence (EI)–the ability to perceive, use, understand, and manage emotions (Mayer et al., 2016), has been concluded to have a positive effect on engagement (Levitats et al., 2019) and a negative effect on the various dimensions of burnout, including exhaustion (e.g., Mérida-López and Extremera, 2017; Extremera et al., 2018). Moreover, emerging research on the role of supervisor EI in employee outcomes (e.g., Miao et al., 2018; Ivcevic et al., 2020) has extended to employee engagement (Levitats et al., 2019), suggesting that supervisor EI acts as a job resource when high and as a demand, when low.

While research focusing on individual-level (employee; supervisor) EI has enriched our understanding of the emergence of engagement and exhaustion; it has de-contextualized these relationships from their organizational setting, downplaying the influence of macro-level EI-related variables, such as organizations’ EI-related culture. We contend that a more contextualized assessment of the EI-related drivers of employee engagement and exhaustion is necessary to further our academic exploration into the EI-related sources of employee engagement and exhaustion. More specifically, there is a need to highlight the top-down influence of perceived EI-related values and practices, on employee wellbeing and supervisor behavior. Such a top-down approach will enable us to explore the extent to which supervisor emotionally intelligent (mis)behavior is shaped by the organization’s EI-related culture, and whether it mediates the relationship between EI-related values and practice and employee engagement and exhaustion.

The lack of relevant contextual EI-related research on the antecedents of engagement and exhaustion, may, in part, be attributed to the absence of suitable measures of EI-related values and practices. Thus, we address the above gaps using a two-study approach. In the first study, we develop measures of EI-related values and human resource management (HRM) practices and test their validity. In the second study, we draw on the job demands-resources (JD-R) framework (Schaufeli and Bakker, 2004; Bakker, 2011; Bakker and Demerouti, 2017), to develop and test a model of the process links between EI-related values and HRM practices and employee engagement and exhaustion, *via* supervisor behavior, and specifically the extent to which it emotionally intelligent.

Our work is inspired by previous calls in the HRM literature for more contextual work on engagement, (Paauwe, 2009); as well as the pioneering work of Jenkins and Delbridge (2013), who explored how contextual features of an organization, such as values and HRM practices, influence management’s ability to promote an internal context which was conducive to the delivery of engagement. We further draw on the Ability-Motivation-Opportunity framework (AMO) (Sterling and Boxall, 2013) to propose that an organization’s EI-supportive culture provides the opportunity (i.e., facilitating conditions) for its supervisors and other members to behave in an emotionally intelligent manner. Thus, we extend this contextual approach by examining the indirect influence of perceived EI-related values and HRM practices on employees’ engagement and exhaustion, *via* their supervisor’s EI-related behavior. By doing so, we aim to make a theoretical and practical contribution to the organizational culture, EI, engagement, and exhaustion literature.

### Engagement, exhaustion, and the job demand-resources model

Work engagement is “a positive, fulfilling, work-related state of mind that is characterized by vigor, dedication, and absorption” (Schaufeli et al., 2002; p. 72). Engaged employees are characterized by: (1) high energy, a willingness to invest effort in work, and persist in the face of difficulties; (2) a sense of significance, enthusiasm, and pride in one’s work; and (3) a state of full concentration in one’s work (Schaufeli and Bakker, 2004). As such, engagement is a positive indicator of employee wellbeing (Schaufeli and Bakker, 2004) and has been empirically demonstrated to be positively associated with high levels of performance, citizenship behavior, and individual wellbeing (Christian et al., 2011; Hakanen and Schaufeli, 2012).

Exhaustion, on the contrary, is the energetic component of burnout–a negative indicator of employee wellbeing. Exhaustion entails feeling of strain, resulting from being depleted of emotional and physical resources (Maslach and Leiter, 2008). While there are varying views on the theoretical compositions of burnout (e.g., Malach-Pines, 2005; Schaufeli and Taris, 2005; Shirom and Melamed, 2006), the construct is most often operationalized as exhaustion, which is viewed as its core element (Toppinen-Tanner et al., 2002; Schaufeli, 2021). As such, this article focuses on the exhaustion component of burnout.

Engagement and exhaustion are often studied using the JD-R model (meta-analyses and reviews: Halbesleben, 2010; Alarcon, 2011; Christian et al., 2011; Lesener et al., 2018). The model was first introduced in the study of burnout (see Demerouti et al., 2001; Bakker et al., 2004), and later extended to work engagement (see Schaufeli and Bakker, 2004; Bakker and Demerouti, 2008). It classifies work characteristics to job demands and resources (Bakker and Demerouti, 2017). Job demands are physical, psychological, social, or organizational aspects of the job that require sustained physical and/or psychological effort and are associated with physiological and/or psychological costs (Demerouti et al., 2001). High job demands evoke a health impairment process, depleting employees from energy, and leading to constant overburdening and exhaustion (Bakker and Demerouti, 2017). Job resources, in contrary, facilitate achieving work goals, reduce job demands and their costs, and stimulate growth and learning (Bakker, 2011). They evoke a motivational process, promoting work engagement, and buffering the harmful impact of job demands (Bakker, 2015; Bakker and Demerouti, 2017; Van Wingerden et al., 2017).

Despite their differences, both resources and demands exist at various levels, including the individual level (i.e., personal resources and demands, such as one’s own high or low EI, respectively), social relations (e.g., supervisor support, team climate), organization of work level (e.g., role clarity, participation in decision making), task level (e.g., autonomy, feedback), and organizational level (e.g., HRM practices, organizational values) (Bakker et al., 2004).

### Emotional intelligence-related organizational culture–Values and practices

Organizational culture is a contextual-level variable that has long been recognized as affecting employees’ attitudes and behavior (Ashkanasy et al., 2000; Aarons and Sawitzky, 2006; Tsai, 2011) as well as organizational effectiveness [see meta-analyses by Hartnell et al. (2019)]. It is a layered construct consisting of deeply held values that translate into normative expectations and behavior (i.e., practices) (Detert et al., 2000; Ployhart et al., 2014). As Trice and Beyer (1984) put it: “Culture has two basic components: (1) its substance, or the networks of meanings contained in its ideologies, norms, and values and (2) its forms, or the practices whereby these meanings are expressed, affirmed and communicated to members” (p. 654).

Ashkanasy (2003a,b) multi-level theory of emotions in organizations stresses that organizational culture is deeply and reciprocally related to organizational members’ emotional views and states. He maintains that just as organizational members’ EI has a bottom-up effect on the organization’s culture and climate, organizational culture has top-down implications for individuals’ emotions and behavior. Building on this model and more recent work on emotions in the workplace (e.g., Ashkanasy and Dorris, 2017; Ashkanasy et al., 2017) and Giorgi’s (2013) proposed model of organizational EI, we propose that a comprehensive framework for understanding the role of EI in organizations should encompass both micro-level individual differences in EI and macro-level dimensions of organizational behavior, such as the presence of an EI-supportive organizational culture.

We use the term EI-supportive organizational culture to refer to the values and practices that signal the importance and instrumentality of emotion-related insights and emotionally intelligent behavior (EIB) within one’s work setting. Our proposed construct builds on existing work about the interaction between emotions and organizational culture. For example, Barsade and Gibson (2007) used the term “affective culture” to suggest that organizations have a unique shared normative system, which shapes the way in which employees express emotions. However, our concept of an EI-supportive organizational culture differs from their approach. Whereas affective culture refers to rules prescribing the appropriateness or inappropriateness of displaying certain emotional expressions in the organization (Barsade et al., 2003; Barsade and Gibson, 2007), the proposed concept of EI-supportive culture refers to a broader normative system, which does not prescribe which emotional expressions are appropriate, but rather embodies the norm about the importance of emotion-related insights and the instrumentality of EIB at work.

We propose that organizations characterized by an EI-supportive culture view EI as an important asset, encouraging members to behave in an emotionally intelligent manner. Like other forms of culture, an organization’s EI-supportive organizational culture is likely to shape its members’ behavior *via* its two main components–values and practices. Values, the invisible part of culture (Quinn and Rohrbaugh, 1983), serve as a built-in normative guide for individuals’ behavior (Roccas and Sagiv, 2017; Jachimowicz et al., 2018), independent of the effect of rewards and punishments as consequences of their actions. Practices are observable behaviors and procedures that are aligned with the organization’s values (Hofstede, 1998). They, too, act as determinants of human behavior, as they signal the instrumentality of behavioral patterns within the organization (Saeed et al., 2019). We explore the meaning of an EI-supportive organizational culture by defining and conceptualizing its components as EI-supportive organizational values and practices.

#### Emotional intelligence-supportive organizational values

Organizational values refer to the general principles that a social group or organization believes are valuable for its objectives and collective welfare. They guide organizational members in their selection or evaluation of behaviors (Bourne and Jenkins, 2013). Regarded as the foundational component of organizational culture, organizational values have long been acknowledged as the normative guide for members’ behavior (e.g., Etzioni, 1961; Fishbein and Ajzen, 1975; Wiener and Vardi, 1990). For example, as early as 1975, the Theory of Planned Behavior (Fishbein and Ajzen, 1975) maintained that individual behavior in organizations should be understood, in part, by considering the internalized normative pressures that arise from the organizations’ values. Since Fishbein and Ajzen’s (1975) seminal work, empirical studies have supported this assumption, providing evidence that organizational culture, and values, in particular, act as a determinant of members’ behavior (e.g., Ye, 2012; Al-Musadieq et al., 2018). Moreover, the person-organization fit literature has demonstrated that in choosing potential employees, organizations test for a correspondence between the candidates’ values and those of the organization (Chatman, 1991; O’Reilly et al., 1991; Kristof-Brown, 2000; Roulin and Krings, 2020).

Emotional intelligence-supportive organizational values convey the importance of emotion-related insights as instrumental. As such, they encourage members to behave in a manner that supports their own emotional wellbeing and that of others by exercising their EI ability to perceive, use, understand, and regulate their emotions. Members of such organizations are likely to be concerned about their own and others’ emotions, including an awareness of the causes and consequences of emotions, the role of emotions in decision-making, and how effective emotion regulation aids in building and maintaining positive interpersonal interactions. At the other end of the continuum are organizations that implicitly and explicitly expect their members to leave their emotions at the door. Members are encouraged to refrain from expressing emotions at work and to make decisions irrespective of their affective implications for colleagues and subordinates. We refer to the values they uphold as “low regard for emotions” values.

The studies on values in the workplace distinguish among four forms of organizational values: attributed (i.e., enacted), espoused, shared, and aspirational (Bourne and Jenkins, 2013). Of them, we attempt to assess the organization’s enacted values, since values are significant to the extent that they may help predict people’s actions, behaviors, and expectations (Maierhofer et al., 2003). Enacted values, unlike the other three forms, may help predict members’ expectations and behaviors for several reasons. First, they reflect the organization’s pattern of past decisions, indicating to employees how to do their jobs (Bourne and Jenkins, 2013; Craft, 2018; Fotaki et al., 2020). Moreover, they are considered to be durable and enduring as they are gradually integrated and strengthened through organizational practices (Bourne et al., 2017). Finally, enacted values determine the selection of organizational goals, consequently influencing the criteria that shape decision-making (Liedtka, 1989). Thus, EI-supportive enacted values may best reflect the collective structure that guides organizational members’ daily decisions and behaviors.

#### Emotional intelligence-supportive organizational practices

For values to be manifested, they are often embedded in organizational life *via* the organization’s practices. Of many forms of organizational practices, such as local management practices and informal social practices (Rousseau and Greller, 1994), formal HRM practices have a vital role in creating and perpetuating organizational culture (Fernández et al., 2003; Marks and Mirvis, 2011; Harrison and Bazzy, 2017). HRM practices are designed to facilitate and support the recruitment, selection, training, and management of employees (Wood and Wall, 2002). They are the primary means by which organizations influence and shape their members’ skills, attitudes, and behavior (Chen and Huang, 2009; Kehoe and Wright, 2013), thereby enforcing the organization’s values.

We conceptualize EI-supportive HRM practices as a component of an EI-supportive organizational culture, which convey the importance of emotions and EIB. In line with Dyer and Holder’s (1988) four-part typology of HRM, we define them as covering: (1) recruitment and selection practices (i.e., screening techniques and tools to assess candidates’ EI-related orientation), (2) performance management (i.e., EI-related employee appraisal and feedback mechanisms), (3) compensation and rewards practices (i.e., monetary and non-monetary rewards that are contingent on EI-related criteria), and (4) training and development practices (i.e., tools aimed at assessing and enhancing members’ emotional wellbeing).

Human resource management practices direct employees’ behavior toward organizational goals (Sun et al., 2007; Takeuchi et al., 2007). We build on this notion to propose that EI-supportive HRM practices will shape organizational members’ behavior. More specifically, we maintain that EI-supportive HRM practices encourage EIB within the organization by signaling the instrumentality of such behavior to candidates and organizational members. For example, it may be argued that once EI-supportive selection and recruitment practices are implemented, interviewers will consciously assess and accept candidates based on how effectively they can perceive, use, understand, and regulate emotions. Similarly, EI-supportive performance appraisal will incline managers and human resource professionals to consider members’ EIB as a desirable criterion when making promotion and compensation decisions. Finally, EI-supportive training and development practices will entail initiatives intended to assess and enhance members’ ability to perceive, understand, utilize, and manage emotions.

### Emotional intelligence-related values and practices and employee engagement and exhaustion

The values and practices through which organizations’ culture is manifested create an internal environment. As such, they may act as job demands or resources, impacting employees’ outcomes (Lopez-Martin and Topa, 2019). Organizations’ EI-supportive HRM practices are likely to act as job resources, as they create organizational aspects of the job that encourage learning and development. Namely, they promote employees’ authentic expression of positive and negative emotions; encourage investment in the development of members’ emotional abilities; reward members for their EIB and provide them with emotional support. Hence, they may stimulate an “upward spiral” (i.e., accumulation of job and personal resources; Salanova et al., 2006), enhancing employee engagement and buffering exhaustion.

By contrast, low regard for emotions values entails job demands. Organizations that adhere to low regard for emotions values are likely to show little to no concern for employees’ emotions. Their members may be expected to refrain from sharing and expressing emotions at work and may not be likely to receive emotional support when confronted with challenges. Hence, it is reasonable that they will experience a depletion of resources and consequently show high exhaustion and low engagement (Bakker and Demerouti, 2017). Considering the above, we hypothesize that:

H1a: Emotional intelligence-supportive HRM practices positively relate to employee engagement. H1b: Emotional intelligence-supportive HRM practices negatively relate to employee exhaustion. H1c: Low regard for emotions values positively relate to employee exhaustion. H1d: Low regard for emotions values negatively relate to employee engagement.

### Supervisor emotionally intelligent (mis)behavior as a job resource (demand) predicting employee engagement and exhaustion

Supervisors’ EI-related behavior may be classified as EIB or emotional misbehavior, depending on the degree to which supervisors successfully exercise the following EI abilities at work: (1) *perceiving emotion*: accurately identifying emotions in oneself, others, and in the environment, (2) *using emotion to facilitate thought*: generating or employing emotions to help thinking and or assist in problem-solving, (3) *understanding emotions*: understanding the causes and consequences of emotions, and (4) *managing emotions*: regulating emotions in oneself and others to achieve desired outcomes by generating and evaluating strategies to influence the course of emotions (Mayer and Salovey, 1997; Mayer et al., 2016).

The EI literature has provided evidence that just as employee EI acts as a personal resource (e.g., Levitats et al., 2019), supervisors’ EIB functions as a job resource (e.g., Ivcevic et al., 2020). Supervisors who exercise high EI abilities contribute to the development of shared goals (George, 2000), and create conditions and opportunities for employees to grow, develop new skills, be promoted, and advance in their careers (Ivcevic et al., 2020). Namely, their ability to perceive and understand emotions and recognize problems and successes may aid them guide employees toward growth opportunities. Moreover, their competence at regulating their own and others’ emotions is likely to help them create the necessary conditions for their subordinates to persist in face of challenges. Acting as a job resource, supervisors’ EIB is likely to predict employees’ engagement and to be negatively associated with exhaustion.

Supervisor emotional misbehavior fails to form a constructive and emotionally enabling work site. Such managers are likely to show little to no concern for their subordinates’ and colleagues’ emotional experience. Instead, they manifest emotional harshness (e.g., berate employees publicly, disregards employees’ emotions) and refrain from regulating their own and others’ emotions (e.g., take out their bad moods on others). By doing so, they become a job demand, creating a downward spiral of additional demands, draining employees’ emotional resources. Thus, we hypothesize that:

H2a: Supervisors’ EIB positively relates to employee engagement. H2b: Supervisors’ EIB negatively relates to employee exhaustion. H2c: Supervisors’ emotional misbehavior positively relate to employee exhaustion. H2d: Supervisors’ emotional misbehavior negatively relates to employee engagement.

### Supervisor’s behavior as a mediator between emotional intelligence-related organizational culture and employee engagement and exhaustion

Supervisors’ behavior may be expected to be largely shaped by the contextual constraints or opportunities created by the organization’s EI-related culture. We draw on the AMO framework (Sterling and Boxall, 2013) to propose that organizational members’ EIB is a product of an interaction between (1) their EI ability (Mayer et al., 2016); (2) their intrinsic motivation to exercise their EI ability, and (3) the opportunity (i.e., extrinsic motivation) created by contextual factors, namely EI-supportive values and HRM practices. Referring to the “opportunity” pillar, we build on previous research on the impact of organizations in shaping employee emotion expression (e.g., O’Neill and Rothbard, 2017), to suggest that the organization’s EI-related culture influences its members’ capacity to respond to emotion-laden situations at work in an emotionally intelligent manner.

Following the above rationale, we assume that an EI-supportive organizational culture creates the contextual support (i.e., opportunity) for supervisors’ EIB. As members realize that the organization recognizes, acknowledges, rewards, and supports certain behaviors, they learn of their importance and instrumentality (Huy, 1999), increasing members’ motivation for such behaviors. Thus, EI-supportive HRM practices may be likely to increase supervisors’ (as well as employees’) motivation and likelihood to behave in an emotionally intelligent manner. Low regard for emotions values, in contrast, signal to members that they should disregard emotions. In such an emotions-dismissive work setting, even knowledgeable, EI-skilled, and intrinsically motivated supervisors may be discouraged to behave in an emotionally intelligent manner. Instead, they will be likely to show emotional misbehavior. Thus, we propose that:

H3a: Emotional intelligence-supportive HRM practices predict supervisor EIB. H3b: Low regard for emotions values predict supervisor emotional misbehavior.

We build upon the rational leading up to H2a–H3b to further hypothesize that:

H4a: Supervisor EIB mediates the relationship between EI-supportive HRM practices and employee engagement. H4b: Supervisor EIB mediates the relationship between EI-supportive HRM practices and employee exhaustion. H4c: Supervisor emotional misbehavior mediates the relationship between low regard for emotions values and employee exhaustion. H4d: Supervisor emotional misbehavior mediates the relationship between low regard for emotions values and employee engagement.

**FIGURE 1:**
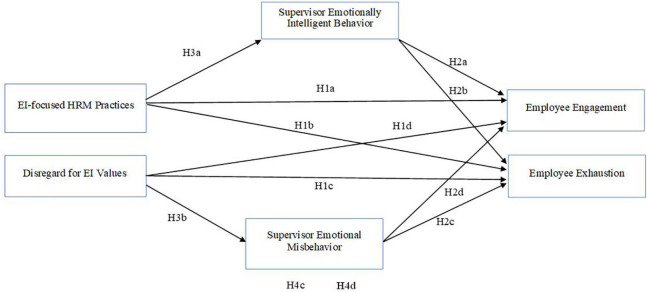
A model of the process links EI-related organizational culture and employee engagement and exhaustion.

The above stated hypotheses and research model are detailed in Figure 1.

## Study 1

The purpose of the first study was to develop and test the psychometric properties of scales assessing EI-related values and practices. We followed a series of qualitative and quantitative phases, to create and validate the scales. Through four phases, we deductively generated a pool of items, conducted exploratory factor analysis to determine if the items adequately captured the two factors, ran confirmatory factor analysis (CFA), and tested for predictive validity.

### Materials and methods

#### Phase 1: Deductive item generation

Drawing from Hinkin’s (1998) deductive approach to item generation, our first goal was to conceptualize EI-related values and HRM practices and to generate corresponding items. First, we defined each construct and generated items by reviewing the literature on organizational values, HRM practices, and EI. The initial item list was reviewed, removing items using the following criteria: (a) relevance: items that did not closely meet the construct’s definition; (b) redundancy: similar items were deleted or combined; (c) clarity; and (d) length: to minimize participant burden, shorter items were preferred.

At the end of this process, the list consisted of 10 items intended to measure perceived EI-related values and practices. We used self-reports, as they were both appropriate and necessary. In the case of values, self-report is needed to assess members’ perceptions of the organization’s enacted values. As for HRM practices, previous research points to the importance of employee perceptions of HRM practices in order to better understand the relationship between HRM practices and employee outcomes (Bowen and Ostroff, 2004; Jose, 2012), since employee perceptions of HRM practices mediate and moderate relationships between an organization’s HRM practices and employees’ attitudes and behaviors (e.g., Liao et al., 2009; Aryee et al., 2012).

#### Participants and procedure

USA workers were recruited through Qualtrics panels. We chose to use Qualtrics panels as they provide samples of workers across industries, thereby increasing the generalizability of the measure (Aguinis and Lawal, 2012). This decision was reinforced by literature proposing that large and diverse samples are recommended for scale development (Clark and Watson, 1995). The final sample included 1,027 working adults over the age of 18 who lived in the United States. About 53.7% of participants were male, 46.5% were managers, 79.4% were White, 10.3% were African American, 1.1% were American Indian or Alaska Native, 0.5% were Native Hawaiian, 4.4% reported Asian American, 1.9% reported biracial or multiracial, and 4.2% reported other. The average age was 40.2 (SD = 13.99).

To reduce the risk of common method bias, we followed two recommendations (Conway and Lance, 2010; Podsakoff et al., 2003, 2012), which may strengthen or weaken relationships between variables. First, all surveys were anonymous, reducing pressure to respond in a socially desirable manner. Second, to ensure that recollection of low regard for emotions values and EI-supportive HRM practices did not influence responses to constructs used for external validity, their measures were placed far apart in the survey.

#### Measures

##### Emotional intelligence-related values and human resource management practices

Ten items were developed to measure EI-related values and practices. Of them, seven items were intended to measure EI-supportive HRM practices, in each of the four domains of HRM practices (see Dyer and Holder, 1988): recruitment and selection practices (e.g., “In hiring interviews, job candidates are asked how they deal with emotions”), performance management (e.g., “In my organization, employees get assessed on how considerate they are to others”), compensation and rewards (e.g., “My organization tends to promote people who connect and relate well with others at work”), and training and development (e.g., “My organization runs workshops to help employees deal with stress”).

In assessing EI-related organizational values, we attempted to reduce response bias by using reverse-scored items (Weijters and Baumgartner, 2012). Thus, we worded these items to measure low regard for emotions values rather than EI-supportive values. We used three items to measure low regard for emotions values. The items covered two domains: dismissing the value and importance of emotions at work (e.g., “In my organization how employees feel matters very little”), and results-above-all task orientation (e.g., “In my organization it is more important to get ahead than to get along”).

For each of the 10 items, participants indicated how often it occurs at work on a scale ranging from 1 (never/almost never) to 6 (always/almost always).

##### Organizational commitment

Commitment to the organization was measured *via* the six-item version of the affective commitment scale (e.g., I feel a strong sense of belonging to my organization; I really feel as if this organization’s problems are my own; Meyer et al., 1993). For each of the six items, participants answered “How often does each of the following happen at your work?” on a seven-point Likert scale, ranging from 1 (strongly disagree) to 6 (strongly agree); (α = 0.77).

##### Engagement

Engagement was assessed using six items on Rich et al.’s (2010) engagement scale. Sample items are “I exert my full effort to my job”; “I feel energetic at my job”; and “At work, I focus a great deal of attention on my job” (α = 0.93).

##### Turnover intentions

Turnover intentions were measured using four items asking participants to describe their job plans, on a seven-point Likert scale, ranging from 1 (strongly disagree) to 6 (strongly agree). The items were: “If an opportunity presented itself, I would pursue another job,” “I have to stay at this job, even though I would rather leave,” “I am only staying in this job because other opportunities are hard to find,” and “I am planning to search for a new job during the next 12 months”; (α = 0.89).

##### Burnout

Burnout was measured using the 10-item short version of the burnout measure (BM) (Malach-Pines, 2005). Participants were asked to indicate how often they experienced various emotions at work (e.g., Disappointed with people, physically weak, tired) on a six-point scale, from “never/almost never” to “always/almost always” (α = 0.96).

### Results

#### Phase 2: Exploratory factor analysis

The main goal of the second phase was to examine the items’ factor structure. The data were analyzed with IBM SPSS Statistics 27. The results are presented in Table 1.

**TABLE 1:** Study 1–Exploratory factor analysis for EI-related organizational culture items (error number in parentheses).

	Factor	
	1	2
My organization invests a lot in making people feel good at work. (e1)	0.93	
My organization tends to promote people who connect and relate well with others at work. (e2)	0.86	
My organization asks about how employees feel at work (e.g., in surveys). (e3)	0.85	
My organization runs workshops to help employees deal with stress. (e4)	0.83	
My organization runs workshops to help employees understand how they can inspire others. (e5)	0.81	
In hiring interviews, job candidates are asked how they deal with emotions. (e6)	0.78	
In my organization, employees get assessed on how considerate they are to others. (e7)	0.75	
In my organization, how employees feel matters very little. (e8)		0.92
In my organization, it is more important to get ahead than to get along. (e9)		0.77
My organization requires employees to leave personal life outside the office. (e10)		0.61

Extraction method: Principal component analysis. Rotation method: Oblimin with Kaiser Normalization. Rotation converged in four iterations.

We conducted principal axis factoring (PAF) with oblique rotation using one-half of the split sample. There are theoretical arguments to assume that an organization’s HRM practices are related to its values. For example, the concept of person-organization fit describes that selection practices aim for a fit between candidates’ values and those of the organization in which they work (Chatman, 1991; O’Reilly et al., 1991; Kristof, 1996).

The decision on the number of factors that had to be retained was based on the examination of the scree plot and the eigenvalues-greater-than-one rule. We identified two factors that explained 70.2% of variance. The first factor included seven items and was interpreted as assessing EI-supportive HRM practices, while the second factor was interpreted as assessing low regard for emotions values.

The EI-supportive HRM practices scale demonstrated high reliability (α = 0.93). The scale mean was 3.49 and items means ranged from 3.27 to 3.74. The scales’ variance was 2.01 and most items captured adequate variance (SD = 1.41). The low regard for emotions values scale demonstrated acceptable reliability (α = 0.71). The scale mean was 3.72 and items means ranged from 3.48 to 3.96. The scales’ variance was 1.63 and most items captured adequate variance (SD = 1.28).

#### Phase 3: Confirmatory factor analysis

The next goal was to conduct a CFA of the two scales. The data were analyzed with IBM SPSS Statistics 27 and IBM SPSS Statistics AMOS 27 (Arbuckle and Wothke, 1999). CFA was conducted using the second half of our sample. In the CFA, the items of low regard for emotions values and EI-supportive HRM practices were modeled as indicators of two related latent variables. Two error terms were allowed to covary based on modification indices. All correlations were conceptually meaningful: e1 and e2 both refer to relationships between employees and e1 and e3 both refer to employees’ wellbeing. After these modifications, fit for the two-factor model was adequate: χ^2^/27 = 4.27, *p* < 0.001, GFI = 0.949, AGFI = 0.912, NFI = 0.959, TLI = 0.955, CFI = 0.968, RMSEA = 0.079, 90% CI [0.066, 0.093[. Standardized factor loadings of disregard for emotions items were acceptable, ranging from 0.58 to 0.79. Standardized factor loadings of EI-supportive HRM practices were high, ranging from 0.73 to 0.90.

#### Phase 4: Predictive validity

To test for predictive validity of the EI-supportive HRM practices and low regard for emotions values scales, we examined whether they predict measures of occupational wellbeing associated with job resources, and negative outcomes associated with emotional demands. We chose to include two measures of occupational wellbeing, for which theoretical and empirical relationships with perceived HRM practices have been demonstrated: commitment to the organization (e.g., Kooij et al., 2010), and engagement (e.g., Alfes et al., 2013; Conway et al., 2016). We further chose to include two negative outcomes often associated with emotional demands: burnout (Livne and Rashkovits, 2018), and turnover intentions (Van der Heijden et al., 2018; Bao and Zhong, 2021).

Multivariate linear regressions were used to test predictive validity of EI-supportive HRM practices and low regard for emotions values using the entire sample, after listwise deletion. The results are presented in Table 2. The two predictors explained 34.9% of the variance in commitment to the organization and 14% of the variance in engagement. EI-supportive HRM practices had a positive and significant association with commitment to the organization (β = 0.67, *p* < 0.001), and engagement (β = 0.36, *p* < 0.001) after controlling for low regard for emotions values. Low regard for emotions values had a negative association with commitment to the organization (β = −0.32, *p* < 0.001), and was not associated with engagement after controlling for EI-supportive HRM practices. EI-supportive HRM practices and low regard for emotions values explained 22.7% of the variance in turnover intentions and 12.7% of the variance in burnout. Low regard for emotions values had a positive association with turnover intentions (β = 0.54, *p* < 0.001), and burnout (β = 0.41, *p* < 0.001) after controlling for EI-supportive HRM practices. EI-supportive HRM practices had a negative association with turnover intentions (β = −0.19, *p* < 0.001), and burnout (β = −0.20, *p* < 0.001) after controlling for low regard for emotions values.

**TABLE 2:** Study 1–Regression analysis for the relationship between EI-supportive HRM practices, low regard for emotions values and the outcome variables (standardized coefficients).

	Positive outcomes						Negative outcomes					
	Organizational commitment			Engagement			Turnover intentions			Burnout		
	β	95% CI		β	95% CI		β	95% CI		β	95% CI	
		Low	Up		Low	Up		Low	Up		Low	Up
EI-Supportive HRM practices	0.68***	0.47	0.55	0.36***	0.25	0.35	−0.19***	−0.23	−0.12	−0.20***	−0.26	−0.13
Low regard for emotions Values	−0.33***	−0.32	−0.23	0.02	−0.04	0.08	0.54***	0.49	0.61	0.41***	0.38	0.52
	*R*^2^ = 0.349***			*R*^2^ = 0.140***			*R*^2^ = 0.227***			*R*^2^ = 0.127***		
	*F*(2,1024) = 274.72***			*F*(2,1024) = 82.93***			*F*(2,1024) = 150.76***			*F*(2,1024) = 74.27***		

***p < 0.001.

### Discussion

Study 1 provided evidence supporting our newly developed scales assessing EI-supportive HRM practices and low regard for emotions values: Both EFA and CFA indicate that EI-supportive HRM practices and low regard for emotions values should be viewed as two separate yet related scales. The reliability of the EI-supportive HRM practices scale was high (α = 0.93) and the low regard for emotions values scale showed acceptable reliability (α = 0.71). As expected, EI-supportive HRM practices predicted commitment to organization and engagement, while low regard for emotions values scale predicted negative work outcomes (i.e., turnover intentions, burnout). These results support the scales’ predictive validity.

The main limitation of Study 1 needs to be pointed out. Since this is the first study to assess EI-supportive HRM practices, there was a discrepancy in the number of items assessing EI-supportive HRM practices and low regard for emotions values (with more items for EI-supportive HRM practices). This may affect the precision of assessing positive and negative HRM practices. Although the measures predicted outcomes in theoretically meaningful ways, offering support for their validity, attention should be paid in future research to developing these measures and testing what they predict in terms of perceived outcomes (e.g., job satisfaction, affective commitment) and objective outcomes (e.g., turnover, absenteeism).

## Study 2

The purpose of the second study was to develop and test a model of the process links between EI-supportive HRM practices and low regard for emotions values, each, with employee engagement and exhaustion, as mediated by supervisors’ emotional (mis)behavior. In doing so, the 14 hypotheses we raised (H1a–H4d) were examined.

### Materials and methods

#### Sample and data collection procedure

Data collection employed Qualtrics panels. Recruitment quotas were created based on the Department of Labor statistics to ensure demographic representativeness of the USA working population. Data were available from all 50 states. Participants were 49.3% male, 50.5% female, and 0.2% reporting “other” gender identities. They self-identified as 83% White/Caucasian, 9.8% Hispanic, 9% Black/African American, 4.4% Asian/Asian American, 2% Biracial or Multiracial, 1.6% American Native or Alaska Native, 0.3% Native Hawaiian or other Pacific Islander, and 2.3% reported other identities. Participants came from all levels in their organization’s hierarchy, with an average of 4.69 (SD = 2.49) on a scale from 0 (entry level) to 10 (head of organization) and reported working on average 37.93 h/week (SD = 15.32). Participants were on average 40.19 years old (SD = 13.93). The results reported below are based on participants who responded to all the study measures (*N* = 12,375).

#### Measures

##### Emotional intelligence-supportive human resource management practices

As in Study 1, EI-supportive HRM practices were assessed by averaging seven items, covering the following five domains: selection, performance review, promotion, training, and general regard for emotions (α = 0.88).

##### Low regard for emotions values

As in Study 1, three items were originally used to measure low regard for emotions values. One of the items was omitted during analysis due to a low factor loading. The remaining two items were “In my organization how employees feel matters very little,” and “In my organization it is more important to get ahead than to get along” (α = 0.61). This Cronbach alpha value is not necessarily indicative of low reliability, given the small number of items (Pallant, 2011). The more appropriate value to report in short scales–inter-item correlation falls in the optimal range of 0.2–0.4 (Briggs and Cheek, 1986) and equals to 0.44, supporting the scale’s reliability.

##### Supervisor emotionally intelligent behavior

The supervisor EIB scale (Ivcevic et al., 2020) was used (see Appendix A). Participants rated their immediate supervisor on 11 items, pertaining to four EI abilities: perceiving emotion (e.g., “My supervisor is good at reading people’s emotions”), using emotions (e.g., “My supervisor generates enthusiasm to motivate others”), understanding emotions (e.g., “My supervisor understands the reasons why employees become upset”), and managing emotions (e.g., “My supervisor keeps calm in difficult situations”). PAF indicated that all items loaded on a single factor (loadings from 0.86 to 0.76; α = 95).

##### Supervisor emotional misbehavior

We developed nine items measuring manifestations of emotional misbehavior, via supervisor harshness (e.g., “My supervisor puts me down me in front of others”) and mismanagement of emotions (e.g., “My supervisor takes out their bad moods on others”). For full list, see Appendix A. PAF indicated a single factor (loadings from 0.87 to 0.72; α = 0.94).

Observer ratings, as opposed to self-reports, were used to assess supervisors’ (mis)behavior, because they seem to be a more valid measure of supervisors’ enacted behavior, which depends both on their ability and motivation for EIB. Supporting validity of observer reports of EI, Elfenbein et al. (2015) found significant consensus across observers’ ratings of a target’s EI, moderate but significant self-observer agreement, and predictive validity for interdependent task performance, even after controlling for cognitive intelligence, personality, trait affect, observer liking, and demographic characteristics.

##### Engagement

We used the six physical engagement items taken from Rich et al.’s (2010) engagement scale (α = 0.90).

##### Exhaustion

Exhaustion was measured using four items from the Maslach Burnout Inventory (e.g., “Working here puts too much stress on me”; Maslach and Jackson, 1981). Participants were asked to rate the items on a six-point scale, from “never/almost never” to “always/almost always” (α = 0.92).

### Results

#### Preliminary analyses

The data were analyzed with IBM SPSS Statistics 27 and IBM Amos 27. To detect the influence of common method bias, we loaded all of the study’s variables onto one factor (common latent factor method). The model accounted for only 30.69% of the variance, which is much lower than the acceptable cut-off of 50% (Fuller et al., 2016). Moreover, the model exhibited very poor fit (see Table 3, one-factor model), providing a good indication that a single factor did not account for the majority of variance in our data.

**TABLE 3:** Study 2–Measurement model comparisons.

Models	χ ^2^ (df)	AGFI	IFI	TLI	CFI	RMSEA	χ ^2^ diff	df diff
Full measurement model, six factors	19342 (682)	0.900	0.946	0.942	0.946	0.047		
Model A, five factors a	34354 (687)	0.800	0.903	0.895	0.903	0.063	15011.409	5***
Model B, five factors b	22152 (687)	0.888	0.938	0.933	0.938	0.050	2809.688	5***
Model C, five factors c	24552 (687)	0.870	0.931	0.926	0.931	0.053	5209.173	5***
Model D, three factors d	104919 (694)	0.446	0.700	0.680	0.700	0.110	85576.326	12***
Model E, one factor e	160941 (697)	0.353	0.539	0.509	0.539	0.136	141598.69	15***

*n* = 12,375, ***p < 0.001. χ^2^, chi-square discrepancy; df, degrees of freedom; AGFI, adjusted goodness of fit; IFI, incremental fit index; TLI, Tucker–Lewis index; CFI, comparative fit index; RMSEA, root mean square error of approximation; χ^2^ diff, difference in chi-square; df diff, difference in degrees of freedom.

^a^EI-supportive HRM practices and supervisors’ emotionally intelligent behavior combined into a single factor; compared to full measurement 6-factor model.

^b^Low regard for emotions values and supervisors’ emotional misbehavior combined into a single factor; compared to full measurement 6-factor model.

^c^EI-supportive HRM practices and low regard for emotions values combined into a single factor; compared to full measurement 6-factor model.

^d^EI-supportive HRM practices, low regard for emotions values, supervisors’ emotionally intelligent behavior, and supervisors’ emotional misbehavior combined into a single factor; compared to full measurement 6-factor model.

^e^Harman’s single factor model; all variables combined into a single factor; compared to full measurement 6-factor model.

#### Measurement model

We carried out a CFA on the full measurement model (six factors), in which all items loaded onto their latent factors as intended. Considering the sample size, the χ^2^ alone is insufficient to determine model fit (Steiger, 2007), so we relied on additional fit indices (Huand Bentler, 1999). The measurement model exhibited good psychometric properties: χ^2^/682 = 28.36, *p* < 0.001, AGFI = 0.900, NFI = 0.944, TLI = 0.942, CFI = 0.946, RMSEA = 0.047, 90% CI [0.046, 0.048[. Five error terms were allowed to covary based on modification indices. All correlations were conceptually meaningful: e6 and e7 both refer to training practices that fall under EI-supportive HRM practices; e33 and e36 both assess engagement by asking about the amount of energy employees invest in their job; e23 and e25 assess supervisors’ emotional misbehavior, by asking about their conduct of criticizing employees; e34 and e35 assess engagement by asking about trying hard to perform one’s job; and e24 and e25 are about supervisor’s insulting behavior in front of others.

#### Measurement model comparisons

To analyze whether all variables in our study were distinct, we carried out a series of nested model comparisons. Specifically, we compared the full measurement model (six factors) comprising all latent variables with a range of alternative models as described in Table 3. Results of sequential χ^2^ difference tests revealed that the model fit of the intended model with six distinct variables was significantly better than all other models (all at *p* < 0.001). This suggests that all study variables were distinct and therefore appropriate for inclusion in the analyses.

#### Test of hypotheses

Table 4 shows descriptive statistics, Cronbach’s alpha coefficients, and zero-order correlations among study variables. The patterns of correlations are consistent with expectations. EI-supportive HRM practices correlated positively with supervisor EIB (*r* = 0.59, *p* < 0.01) and employee engagement (*r* = 0.11, *p* < 0.01) and negatively with employee exhaustion (*r* = −0.32, *p* < 0.01). Low regard for emotions values correlated positively with supervisor emotional misbehavior (*r* = 0.46, *p* < 0.01) and employee exhaustion (*r* = 0.43, *p* < 0.01) and negatively with employee engagement (*r* = −0.04, *p* < 0.01). Furthermore, supervisor EIB correlated positively with employee engagement (*r* = 0.18, *p* < 0.01) and negatively with employee exhaustion (*r* = −0.40, *p* < 0.01). Supervisor emotional misbehavior correlated positively with employee exhaustion (*r* = 0.45, *p* < 0.01) and negatively with employee engagement (*r* = −0.04, *p* < 0.01).

**TABLE 4:** Study 2–Descriptive statistics and zero-order correlations (*N* = 12,375).

	Mean	SD	1	2	3	4	5	6
EI-supportive HRM practices	3.24	1.24	(0.88)					
Low regard for emotions values	3.23	1.35	−0.15**	(0.61)				
Supervisor emotionally intelligent behavior	3.55	1.22	0.59**	−0.32**	(0.95)			
Supervisor emotional misbehavior	1.90	1.16	−0.03**	0.46**	−0.28**	(0.94)		
Engagement	4.80	0.98	0.11**	−0.04**	0.18**	−0.04**	(0.90)	
Exhaustion	3.12	1.38	−0.32**	0.43**	−0.40**	0.45**	0.05**	(0.92)

Values in parentheses in the diagonal are Cronbach alpha coefficients for each measure.

**p < 0.01 (two-tailed).

The study’s hypotheses were tested using Structural Equation Models (SEM) on AMOS 27.0 software. SEM allows us to test complicated mediation models, which include multiple independent variables, mediators or outcomes, including latent constructs, in a single analysis. Thus, it allows for ease of interpretation and estimation, while simplifying the testing of mediation hypotheses (MacKinnon, 2012). Model fit was high: χ^2^/686 = 30.49, *p* < 0.001, GFI = 0.905, AGFI = 0.892, NFI = 0.942, TLI = 0.937, CFI = 0.942, RMSEA = 0.049, 90% CI [0.048, 0.049 [, providing preliminary support for our model and hypotheses.

### The relationship between emotional intelligence-supportive human resource management practices, low regard for emotions values and employee engagement and exhaustion

Table 5 presents standardized regression weight estimates relating the model’s independent variables, mediators, and dependent variables. EI-supportive HRM practices predicted employee engagement (β = 0.15, *p* < 0.01), and were negatively related to employee exhaustion (β = −0.27, *p* < 0.001), lending support for H1a and H1b, respectively. Consistent with H1c, low regard for emotions values predicted employee exhaustion (β = 0.44, *p* < 0.01). However, contrary to H1d, low regard for emotions values predicted employee engagement (β = 0.03, *p* < 0.05). Considering the small effect size, it seems that although statistically significant, this positive relationship is not meaningful and is solely due to our large sample size.

**TABLE 5:** Study 2–Regression analysis for the relationships between independent variables, mediators, and dependent variables (standardized coefficients).

	Mediators						Outcomes					
	Supervisor emotionally intelligent behavior			Supervisor emotional misbehavior			Engagement			Exhaustion		
	β	95% CI		β	95% CI		β	95% CI		β	95% CI	
		Low	Up		Low	Up		Low	Up		Low	Up
EI-supportive HRM practices	0.69**	0.68	0.70				0.15**	0.13	0.17	−0.25**	−0.27	−0.22
Low regard for emotions values				0.51**	0.47	0.53	0.03*	0.00	0.06	0.44**	0.40	0.47
Supervisor emotionally intelligent behavior							0.23**	0.20	0.26	−0.07**	−0.10	−0.05
Supervisor emotional misbehavior							0.01	−0.01	0.04	0.30**	0.28	0.33

*p < 0.05; **p < 0.01.

### The relationship between supervisor behavior and employee engagement and exhaustion

As given in Table 5, supervisor’s EIB positively related to employee engagement (β = 0.23, *p* < 0.01) and negatively related to employee exhaustion (β = −0.07, *p* < 0.01), lending support for H2a and H2b. Supervisor emotional misbehavior positively related to employee exhaustion (β = 0.30, *p* < 0.01) and was not associated to employee engagement, supporting H2c but not H2d.

### The relationship between emotional intelligence-supportive human resource management practices, low regard for emotions values, and supervisor behavior

As hypothesized, EI-supportive HRM practices positively related to supervisor EIB (β = 0.69, *p* < 0.01), whereas low regard for emotions values positively related to supervisor emotional misbehavior (β = 0.51, *p* < 0.01). These results support H3a and H3b.

### Mediation effects of supervisor emotionally intelligent behavior on the relationships between emotional intelligence-supportive human resource management practices and employee engagement and exhaustion

**FIGURE 2:**
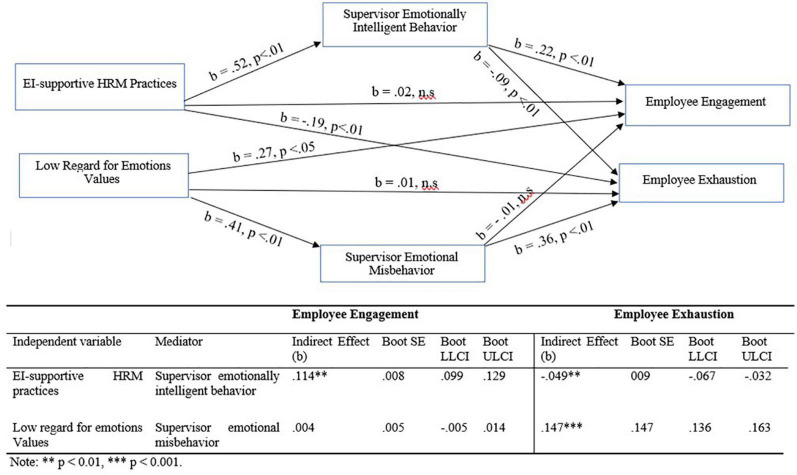
Mediation effects of supervisor emotionally intelligent behavior and supervisor emotional misbehavior on the relationship of EI-supportive HRM practices and low regard for emotions values with employee engagement and exhaustion.

The AMOS mediation regression weight estimates (standardized) are provided in Table 6. Consistent with H4a, bootstrap mediation analysis showed that the relationship between the EI-supportive HRM practices and employee engagement was mediated by supervisor EIB (β = 0.16, *p* = 0.003; CI: 0.14, 0.18). Whereas the total effect of EI-supportive HRM practices was significant (β = 0.15, *p* < 0.01), the direct effect was insignificant. Thus, supervisor EIB fully mediated the relationship between EI-supportive HRM practices and employee engagement, supporting H4a (see Figure 2). Consistent with H4b, supervisor EIB partially mediated the relationship between EI-supportive HRM practices and employee exhaustion (β = −0.05, *p* = 0.004, CI: −0.06, −0.03). As presented in Table 6, the total effect of EI-supportive HRM practices (β = −0.24, *p* < 0.01) was higher than its direct effect (β = −0.20, *p* < 0.01).

**TABLE 6:** Mediating effect of supervisor emotionally intelligent behavior and supervisor emotional misbehavior on the relationships between EI-supportive HRM practices and low regard for emotions values and employee engagement and exhaustion.

IV^a^	Med^b^	DV^c^	IV → Med A	Med → DV B	IV → DV c’	Total C
EI-supportive HRM practices	→ Supervisor emotionally intelligent behavior	→ Employee engagement	0.69** (0.01)	0.23* (0.01)	−0.014 (0.01)	0.15** (0.01)
EI-supportive HRM practices	→ Supervisor emotionally intelligent behavior	→ Employee exhaustion	0.69** (0.01)	−0.07* (0.01)	−0.20** (0.01)	−0.24** (0.01)
Low regard for emotions values	→ Supervisor emotional misbehavior	→ Employee engagement	0.51** (0.02)	0.01 (0.01)	0.03 (0.02)	0.03* (0.01)
Low regard for emotions values	→ Supervisor emotional misbehavior	→ Employee exhaustion	0.51** (0.02)	0.30* (0.01)	0.29* (0.01)	0.44** (0.02)

Numbers in cells are standardized coefficients–β, SE (β) in parentheses.

^a^IV, independent variable; ^b^Med, mediator; ^c^DV, dependent variable.

*p < 0.05; **p < 0.01.

### Mediation effect of supervisor emotional misbehavior on the relationships between emotional intelligence-supportive human resource management practices and employee engagement and exhaustion

As Table 6 presents, the process links between low regard for emotions values and exhaustion, *via* supervisor emotional misbehavior was significant (β = 0.16, *p* = 0.002; CI: 0.14, 0.17). While the total effect of low regard for emotions values on exhaustion was significant (β = 0.44, *p* < 0.001), the direct effect of low regard for emotions values was significantly lower (β = 0.29, *p* < 0.05). Thus, supervisor emotional misbehavior partially mediated the relationship between the low regard for emotions values and employee exhaustion, supporting H4c. In the contrary, supervisor emotional misbehavior did not mediate the relationship between low regard for emotions values and employee engagement. The process links between low regard for emotions values and engagement, *via* supervisor emotional misbehavior were insignificant. Thus, H4d was not supported.

### Discussion

This study tested a model describing the process links between EI-supportive HRM practices, low regard for emotions values and employee engagement and exhaustion, *via* supervisor EI-related behavior. EI-supportive HRM practices added to the explanation of supervisor EIB, whereas low regard for emotions values predicted supervisor emotional misbehavior. Supervisor EIB fully mediated the relationship between EI-supportive HRM practices and employee engagement and partially mediated the relationship between EI-supportive HRM practices and employee exhaustion. Supervisor emotional misbehavior partially mediated the relationship between low regard for emotions values and employee exhaustion but did not mediate its relationship with employee engagement. The above results represent a contribution to research on employee engagement and exhaustion, as well as to the EI literature. In the following sections, we discuss the implications of our findings for theory, managerial and human resource practice, address the meaning of these results in respect to previous work in this field, discuss the limitations and strengths of this study and propose a direction for future research.

## General discussion

### Theoretical and practical implications

This two-study research makes several methodological, empirical, theoretical, and practical contributions. On a methodological note, EI-related values and practices were proposed as aspects of EI-related culture, and respective measurement scales were developed and validated (study 1). Despite their limitations (as detailed below), these newly developed measures may aid EI scholars in future research on the role of contextual EI-related variables in predicting employee and organizational outcomes.

As for empirical contribution, though our study is limited due to its cross-sectional design, it provides initial evidence of the process links between EI-related values and practices, supervisor emotional (mis)behavior and employee engagement and exhaustion. Such findings concerning supervisors’ behavior as a mediator between contextual factors and employees’ individual level outcomes extend on previous research relating supervisor EIB to additional resources (e.g., Ivcevic et al., 2020). Moreover, they corroborate Levitats et al., 2019 proposition that supervisors’ EI-related behavior is a “double edged sword,” such that EIB acts as a job resource whereas emotional misbehavior is a hindrance demands. Finally, these findings support previous claims that supervisors are a powerful source of emotion contagion (Li et al., 2019), such that their emotionally intelligent (mis)behavior predict subordinates’ affect-related outcomes of engagement and exhaustion.

A related theoretical contribution emerges from our findings concerning the distinct mediation effect of supervisor behavior on the relationship between organization-level resources vs. demands (i.e., EI-supportive HRM practices and low regard for emotions values) on employee engagement and exhaustion, respectively. Whereas supervisor EIB fully mediated the relationship between EI-supportive HRM practices and engagement, the mediation effect of supervisor emotional misbehavior on the relationship between low regard for emotions values and exhaustion was partial. Moreover, the effect of the “negative” EI-related variables (low regard for emotions values, supervisor emotional misbehavior) on exhaustion was stronger than those of the “positive” EI-related variables (EI-supportive HRM practices, supervisor EIB) on engagement. These findings echo research showing that EI ability might be more successful in predicting fewer negative outcomes than more positive outcomes (Brackett et al., 2004). Furthermore, it corroborates the proposition that negative is more powerful than positive–negative events and emotions have a stronger and long-lasting impact on negative outcomes than positive events or emotions have on positive outcomes (Baumeister et al., 2001).

On a related note, in terms of theory development, our work contributes to the EI, engagement, and exhaustion literature. Unlike previous studies (Mérida-López and Extremera, 2017; Extremera et al., 2018), which investigated the effect of employees’ EI on their engagement and exhaustion, we highlight the impact of EI-related organizational culture and supervisor behavior on employee engagement and exhaustion. Borrowing from JD-R model (Bakker and Demerouti, 2017), our findings suggest that EI-supportive HRM practices and supervisor EIB act as job resources, augmenting employee engagement, whereas low regard for emotions values and supervisor emotional misbehavior act as hindrance demands, contributing to higher levels of employee exhaustion. Furthermore, our findings of a top-down effect of EI-related values and practices on supervisors’ behavior and employees’ engagement and exhaustion support the AMO framework (Sterling and Boxall, 2013), as they suggest that a strong organization’s EI-supportive culture provides its member with the opportunity to behave in an emotionally intelligent manner, whereas organizations that uphold low regard for emotions may inhibit their members’ EIB.

On a practical level, our findings provide HR professionals with insights for enhancing employee engagement and mitigating exhaustion. As our findings suggest, EI-supportive HRM practices and supervisors’ EIB may be used to enhance employee engagement, whereas low regard for emotions values and supervisor emotional misbehavior promote employee exhaustion. It may therefore be concluded that organizations will benefit from enforcing EI-supportive HRM practices, imposing codes against emotional misbehavior and investing in EI training for their members. Recent meta-analyses show that EI can be trained (Hodzic et al., 2018; Mattingly and Kraiger, 2019), offering a viable route for how HR policies can build leadership resources as part of organizational training programs.

### Limitations and future research

While this study advances our understanding of the role of individual and organizational EI-related factors and their downstream outcomes, it also has several limitations. The most prominent is the cross-sectional nature of the data, which prevents us from inferring causality. In the absence of empirical evidence concerning the direction of causality in these relations, for model testing purposes, we made judgments based on our current understanding of the constructs involved. For the relationship between the model’s independent and mediating variables, we reasoned that EI-related culture is a broader construct than supervisor behavior, and is therefore more likely to influence supervisor behavior than vice versa. However, although a reverse causal ordering between HRM practices and supervisor behavior would not be theoretically meaningful, we cannot rule it out. Thus, future research should use a longitudinal design to examine if and how the EI-related behavior of new recruits (supervisors) changes through time, as they learn about organizations’ culture. Moreover, a longitudinal will be beneficial in establishing a causal relationship between supervisors’ EI-related behavior and employee engagement and exhaustion.

A second limitation concerns the nature of our sampling method. While culture is an organizational level variable, it was examined at the individual level, examining how organizational members’ perception of EI-related values and practices relate to engagement and exhaustion. This does not invalidate our results. However, future research should use a sampling method better suited to examining EI-related organizational culture at the collective level. Similarly, our data concerning supervisors’ EI-related behavior was obtained from individual employees. Ideally, researchers would obtain reports from multiple subordinates of the same supervisor and aggregate their data for greater reliability. Having reports across members of the same team will enable using random coefficient modeling (Goldstein, 1987), with employees nested within their supervisor’s team.

## Conclusion

Notwithstanding the above limitations, this two-study research points to a need to approach EI from a macro-level perspective of organizational culture. We introduced the theoretical concept of an EI-supportive organizational culture and developed measures of perceived EI-supportive HRM practices and low regard for emotions values. Our results set preliminary foundations for the theoretical discussion and empirical work on the joint role of organizational EI-supportive factors in creating the opportunity for members’ EIB, thereby predicting employees’ work experiences. We hope that this work will inspire additional theoretical development and the empirical testing to advance scholars’ and practitioners’ understanding of the dynamic interplay between the multi-level affective factors that shape individuals’ attitudes and behavior in organizations.

## Data availability statement

The raw data supporting the conclusions of this article will be made available by the authors, without undue reservation.

## Ethics statement

The studies involving human participants were reviewed and approved by Institutional Review Board at Yale University. The patients/participants provided their written informed consent to participate in this study.

## Author contributions

ZL and ZI contributed to the conception and design of the study. ZI organized the database. ZL performed the statistical analysis and wrote the first draft of the manuscript. All authors contributed to manuscript revision, read, and approved the submitted version.

## Appendix A

**TABLE A1:**

Sub-component	Supervisor emotionally intelligent behavior items
Perceive	If someone is feeling upset about a decision, my supervisor will notice.
	My supervisor realizes when people are dissatisfied at work.
	My supervisor is good at reading peoples’ emotions.
Use	My supervisor helps people find ways to channel their dissatisfaction into making a productive change.
	My supervisor encourages people to use their curiosity to learn and come up with ideas.
	My supervisor generates enthusiasm to motivate others.
	My supervisor learns from both disappointments and successes when planning for the future.
Understand	My supervisor understands the reasons why employees become upset.
	My supervisor understands how their decisions and behaviors affect how others feel at work.
Manage	My supervisor keeps calm in difficult situations.
	My supervisor is good at helping others feel better when they are disappointed or upset.

**Sub-component**	**Supervisor emotional misbehavior items**

Harshness	My supervisor takes my work achievements and passes them off as their own.
	My supervisor criticizes me harshly.
	My supervisor speaks badly about me behind my back.
	My supervisor puts me down me in front of others.
Mismanagement of emotions	My supervisor displays uncontrolled anger.
	My supervisor has emotional outbursts.
	My supervisor takes out their bad moods on others.
	My supervisor lets their emotions get out of control.
	I am afraid of being around my supervisor when they are in a bad mood.

## References

[B1] Aarons G. A. Sawitzky A. C. (2006). Organizational climate partially mediates the effect of culture on work attitudes and staff turnover in mental health services. *Adm. Policy Ment. Health Ment. Health Serv. Res.* 33 289–301. 10.1007/s10488-006-0039-1 16544205 PMC1564125

[B2] Aguinis H. Lawal S. O. (2012). Conducting field experiments using eLancing’s natural environment. *J. Bus. Ventur.* 27 493–505. 10.1016/j.jbusvent.2012.01.002

[B3] Alarcon G. M. (2011). A meta-analysis of burnout with job demands, resources, and attitudes. *J. Vocat. Behav.* 79 549–562. 10.1016/j.jvb.2011.03.007

[B4] Alfes K. Shantz A. D. Truss C. Soane E. C. (2013). The link between perceived human resource management practices, engagement and employee behaviour: A moderated mediation model. *Int. J. Hum. Resour. Manage.* 24 330–351. 10.1080/09585192.2012.679950

[B5] Al-Musadieq M. Nurjannah N. Raharjo K. Solimun S. Fernandes A. A. R. (2018). The mediating effect of work motivation on the influence of job design and organizational culture against HR performance. *J. Manage. Dev.* 37 452–469. 10.1108/jmd-07-2017-0239

[B6] Arbuckle J. L. Wothke W. (1999). *Amos 4.0 user’s guide*. Chicago, IL: SmallWaters Corporation.

[B7] Aryee S. Walumbwa F. O. Seidu E. Y. M. Otaye L. E. (2012). Impact of high-performance work systems on individual- and branch-level performance: Test of a multilevel model of intermediate linkages. *J. Appl. Psychol.* 97 287–300. 10.1037/a0025739 21967297

[B8] Ashkanasy N. M. (2003a). “Emotions in organizations: A multi-level perspective,” in *Research in multi-level issues*, Vol. 2, eds Yammarino F. J. Dansereau F. (Oxford: Elsevier Science), 9–54.

[B9] Ashkanasy N. M. (2003b). “Emotions at multiple levels: An integration,” in *Research in multi-level issues*, Vol. 2 eds Dansereau F. Yammarino F. J. (Oxford: Elsevier Science), 71–81.

[B10] Ashkanasy N. M. Dorris A. D. (2017). Emotions in the workplace. *Annu. Rev. Organ. Psychol. Organ. Behav.* 4 67–90.

[B11] Ashkanasy N. M. Troth A. C. Lawrence S. A. Jordan P. J. (2017). “Emotions and emotional regulation,” in *Research in personnel and human resources management*, Vol. 35 eds Buckley M. R. Halbesleben J. R. B. Wheeler A. R. (Bingley: Emerald Group Publishing).

[B12] Ashkanasy N. M. Wilderom C. P. Peterson M. F. (eds) (2000). *Handbook of organizational culture and climate.* Thousand Oaks, CA: Sage.

[B13] Bakker A. B. (2011). An evidence-based model of work engagement. *Curr. Dir. Psychol. Sci.* 20 265–269.

[B14] Bakker A. B. (2015). “Top-down and bottom-up interventions to increase work engagement,” in *APA handbook of career intervention*, eds Hartung P. J. Savickas M. L. Walsh W. B. (Washington, DC: American Psychological Association). 427–438. 10.1037/14439-031

[B15] Bakker A. B. Demerouti E. (2008). Towards a model of work engagement. *Career Dev. Int.* 13 209–223. 10.1108/13620430810870476

[B16] Bakker A. B. Demerouti E. (2017). Job demands–resources theory: Taking stock and looking forward. *J. Occup. Health Psychol.* 22 273–285. 10.1037/ocp0000056 27732008

[B17] Bakker A. B. Demerouti E. Verbeke W. (2004). Using the job demands-resources model to predict burnout and performance. *Hum. Resour. Manage.* 43 83–104. 10.1002/hrm.20004

[B18] Bao Y. Zhong W. (2021). Public service motivation matters: Examining the differential effects of challenge and hindrance stressors on organizational identification and turnover intention. *Public Manage. Rev.* 23 545–566. 10.1080/14719037.2019.1699944

[B19] Barsade S. G. Gibson D. E. (2007). Why does affect matter in organizations? *Acad. Manage. Perspect.* 21 36–59. 10.5465/amp.2007.24286163

[B20] Barsade S. G. Brief A. P. Spataro S. E. (2003). “The affective revolution in organizational behavior: The emergence of a paradigm,” in *Organizational behavior: The state of the science*, ed. Greenberg J. (Mahwah, NJ: Lawrence Erlbaum Associates Publishers), 3–52.

[B21] Baumeister R. F. Bratslavsky E. Finkenauer C. Vohs K. D. (2001). Bad is stronger than good. *Rev. Gen. Psychol.* 5 323–370. 10.1037/1089-2680.5.4.323

[B22] Bourne H. Jenkins M. (2013). Organizational values: A dynamic perspective. *Organ. Stud.* 34 495–514. 10.1177/0170840612467155

[B23] Bourne H. Jenkins M. Parry E. (2017). Mapping espoused organizational values. *J. Bus. Ethics* 159 133–148. 10.1007/s10551-017-3734-9

[B24] Bowen D. E. Ostroff C. (2004). Understanding HRM–firm performance linkages: The role of the “Strength” of the HRM System. *Acad. Manage. Rev.* 29 203–221. 10.5465/amr.2004.12736076

[B25] Brackett M. A. Mayer J. D. Warner R. M. (2004). Emotional intelligence and its relation to everyday behaviour. *Pers. Individ. Dif.* 36 1387–1402. 10.1016/S0191-8869(03)00236-8

[B26] Brackett M. A. Palomera R. Mojsa-Kaja J. Reyes M. R. Salovey P. (2010). Emotion-regulation ability, burnout, and job satisfaction among British secondary-school teachers. *Psychol. Sch.* 47 406–417. 10.1002/pits.20478

[B27] Briggs S. R. Cheek J. M. (1986). The role of factor analysis in the development and evaluation of personality scales. *J. Pers.* 54 106–148. 10.1111/j.1467-6494.1986.tb00391.x

[B28] Chatman J. A. (1991). Matching people and organizations: Selection and socialization in public accounting firms. *Adm. Sci. Q.* 36 459–484. 10.2307/2393204

[B29] Chen C. J. Huang J. W. (2009). Strategic human resource practices and innovation performance—The mediating role of knowledge management capacity. *J. Bus. Res.* 62 104–114. 10.1016/j.jbusres.2007.11.016

[B30] Christian M. S. Garza A. S. Slaughter J. E. (2011). Work Engagement: A quantitative review and test of its relations with task and contextual performance. *Pers. Psychol.* 64 89–136. 10.1111/j.1744-6570.2010.01203.x

[B31] Clark L. A. Watson D. (1995). Constructing validity: Basic issues in objective scale development. *Psychol. Assess.* 7 309–319. 10.1037/1040-3590.7.3.309 PMC6754793 30896212

[B32] Conway E. Fu N. Monks K. Alfes K. Bailey C. (2016). Demands or resources? The relationship between HR practices, employee engagement, and emotional exhaustion within a hybrid model of employment relations. *Hum. Resour. Manage.* 55 901–917. 10.1002/hrm.21691

[B33] Conway J. M. Lance C. E. (2010). What reviewers should expect from authors regarding common method bias in organizational research. *J. Bus. Psychol.* 25 325–334. 10.1007/s10869-010-9181-6

[B34] Craft J. L. (2018). Common thread: The impact of mission on ethical business culture. A case study. *J. Bus. Ethics* 149 127–145. 10.1007/s10551-016-3034-9

[B35] Demerouti E. Bakker A. B. Nachreiner F. Schaufeli W. B. (2001). The job demands-resources model of burnout. *J. Appl. Psychol.* 86 499–512. 10.1037/0021-9010.86.3.499? 11419809

[B36] Detert J. R. Schroeder R. G. Mauriel J. J. (2000). A framework for linking culture and improvement initiatives in organizations. *Acad. Manage. Rev.* 25 850–863. 10.2307/259210

[B37] Dyer L. Holder G. (1988). “Toward a strategic perspective of human resource management,” in *Human resource management: Evolving roles and responsibilities, ASPA/BNA Handbook of Human Resource Management*, Vol. I. ed. Dyer L. (Washington, DC: Bureau of National Affairs).

[B38] Elfenbein H. A. Barsade S. G. Eisenkraft N. (2015). The social perception of emotional abilities: Expanding what we know about observer ratings of emotional intelligence. *Emotion* 15 17–34. 10.1037/a0038436 25664949

[B39] Etzioni A. (1961). *A comparative analysis of complex organizations: On power, involvement, and their correlates*. New York, NY: Free Press of Glencoe.

[B40] Extremera N. Mérida-López S. Sánchez-Álvarez N. Quintana-Orts C. (2018). How does emotional intelligence make one feel better at work? The mediational role of work engagement. *Int. J. Environ. Res. Public Health* 15:1909. 10.3390/ijerph15091909 30200548 PMC6164137

[B41] Fernández E. Junquera B. Ordiz M. (2003). Organizational culture and human resources in the environmental issue: A review of the literature. *Int. J. Hum. Resour. Manage.* 14 634–656. 10.1080/0958519032000057628

[B42] Fishbein M. Ajzen I. (1975). *Belief, attitude, intention and behavior: An introduction to theory and research*. Reading, MA: Addison-Wesley.

[B43] Fotaki M. Lioukas S. Voudouris I. (2020). Ethos is destiny: Organizational values and compliance in corporate governance. *J. Bus. Ethics* 166 19–37. 10.1007/s10551-019-04126-7

[B44] Fuller C. M. Simmering M. J. Atinc G. Atinc Y. Babin B. J. (2016). Common methods variance detection in business research. *J. Bus. Res.* 69 3192–3198. 10.1038/s41467-022-31007-x 35725563 PMC9209422

[B45] George J. M. (2000). Emotions and leadership: The role of emotional intelligence. *Hum. Relat.* 53 1027–1055. 10.1177/0018726700538001

[B46] Giorgi G. (2013). Organizational emotional intelligence: Development of a model. *Int. J. Organ. Anal.* 21 4–18. 10.1108/19348831311322506

[B47] Goldstein H. (1987). *Multilevel models in education and social research.* Oxford Oxford University Press.

[B48] Hakanen J. J. Schaufeli W. B. (2012). Do burnout and work engagement predict depressive symptoms and life satisfaction? A three-wave seven-year prospective study. *J. Affect. Disord.* 141 415–424. 10.1016/j.jad.2012.02.043 22445702

[B49] Halbesleben J. R. B. (2010). “A meta-analysis of work engagement: Realtionships with burnout, demands, resources and consequences” in *Work engagement: A handbook of essential theory and research*, eds Bakker A. B. Leiter M. P. (New York, NY: Psychology Press), 102–117.

[B50] Harrison T. Bazzy J. D. (2017). Aligning organizational culture and strategic human resource management. *J. Manage. Dev.* 36 1260–1269. 10.1108/JMD-12-2016-0335

[B51] Hartnell C. A. Ou A. Y. Kinicki A. J. Choi D. Karam E. P. (2019). A meta-analytic test of organizational culture’s association with elements of an organization’s system and its relative predictive validity on organizational outcomes. *J. Appl. Psychol.* 104 832–850. 10.1037/apl0000380 30628804

[B52] Hinkin T. R. (1998). A brief tutorial on the development of measures for use in survey questionnaires. *Organ. Res. Methods* 1 104–121. 10.1177/109442819800100106

[B53] Hodzic S. Scharfen J. Ripoll P. Holling H. Zenasni F. (2018). How efficient are emotional intelligence trainings: A meta-analysis. *Emot. Rev.* 10 138–148. 10.1177/1754073917708613

[B54] Hofstede G. (1998). Attitudes, values and organizational culture: Disentangling the concepts. *Organ. Stud.* 19 477–493. 10.1177/017084069801900305

[B55] Hu L. Bentler P. M. (1999). Cutoff criteria for fit indexes in covariance structure analysis: Conventional criteria versus new alternatives. *Struct. Equ. Modeling* 6 1–55. 10.1080/10705519909540118

[B56] Huy Q. N. (1999). Emotional capability, emotional intelligence, and radical change. *Acad. Manage. Rev.* 24 325–345. 10.5465/amr.1999.1893939

[B57] Ivcevic Z. Moeller J. Menges J. Brackett M. A. (2020). Supervisor emotionally intelligent behavior and employee creativity. *J. Creat. Behav.* 55 79–91. 10.1002/jocb.436

[B58] Jachimowicz J. M. Hauser O. P. O’Brien J. D. Sherman E. Galinsky A. D. (2018). The critical role of second-order normative beliefs in predicting energy conservation. *Nat. Hum. Behav.* 2 757–764. 10.1038/s41562-018-0434-0 31406290

[B59] Jenkins S. Delbridge R. (2013). Context matters: Examining ‘soft’and ‘hard’approaches to employee engagement in two workplaces. *Int. J. Hum. Resour. Manage.* 24 2670–2691. 10.1080/09585192.2013.770780

[B60] Jose G. (2012). Satisfaction with HR practices and employee engagement: A social exchange perspective. *J. Econ. Behav. Stud.* 4 423–430. 10.22610/jebs.v4i7.343

[B61] Kehoe R. R. Wright P. M. (2013). The impact of high-performance human resource practices on employees’ attitudes and behaviors. *J. Manage.* 39 366–391. 10.1177/0149206310365901

[B62] Kooij D. T. Jansen P. G. Dikkers J. S. De Lange A. H. (2010). The influence of age on the associations between HR practices and both affective commitment and job satisfaction: A meta-analysis. *J. Organ. Behav.* 31 1111–1136. 10.1002/job.666

[B63] Kristof A. L. (1996). Person-organization fit: An integrative review of its conceptualizations, measurement, and implications. *Pers. Psychol.* 49 1–49. 10.1111/j.1744-6570.1996.tb01790.x

[B64] Kristof-Brown A. L. (2000). Perceived applicant fit: Distinguishing between recruiters’ perceptions of person-job and person-organization fit. *Pers. Psychol.* 53, 643–671. 10.1111/j.1744-6570.2000.tb00217.x

[B65] Lesener T. Gusy B. Wolter C. (2018). The job demands-resources model: A meta-analytic review of longitudinal studies. *Work Stress* 33 76–103. 10.1080/02678373.2018.1529065

[B66] Levitats Z. Vigoda-Gadot E. Vashdi D. R. (2019). Engage them through emotions: Exploring the role of emotional intelligence in public sector engagement. *Public Adm. Rev.* 79 841–852. 10.1111/puar.13113

[B67] Li Y. N. Law K. S. Yan M. (2019). Other-caring or other-critical? A contagious effect of leaders’ emotional triads on subordinates’ performance. *Asia Pac. J. Manage.* 36 995–1021. 10.1007/s10490-018-9617-5

[B68] Liao H. Toya K. Lepak D. P. Hong Y. (2009). Do they see eye to eye? Management and employee perspectives of high-performance work systems and influence processes on service quality. *J. Appl. Psychol.* 94 371–391. 10.1037/a0013504 19271796

[B69] Liedtka J. M. (1989). Value congruence: The interplay of individual and organizational value systems. *J. Bus. Ethics* 8 805–815. 10.1007/BF00383780

[B70] Livne Y. Rashkovits S. (2018). Psychological empowerment and burnout: Different patterns of relationship with three types of job demands. *Int. J. Stress Manage.* 25 96–108. 10.1037/str0000050

[B71] Lopez-Martin E. Topa G. (2019). Organizational culture and job demands and resources: Their impact on employees’ wellbeing in a multivariate multilevel model. *Int. J. Environ. Res. Public Health* 16:3006. 10.3390/ijerph16173006 31438459 PMC6747151

[B72] MacKinnon D. P. (2012). *Introduction to statistical mediation analysis*. New York, NY: Routledge.

[B73] Maierhofer N. I. Rafferty A. E. Kabanoff B. (2003). “When and why are values important in organizations?” in *Emerging perspectives on values in organizations*, eds Gilliland S. W. Steiner D. D. Skarlicki D. P. (Greenwich, CT: Information Age Publishing), 3–32.

[B74] Malach-Pines A. (2005). The burnout measure, short version. *Int. J. Stress Manage.* 12 78–88. 10.1037/1072-5245.12.1.78

[B75] Marks M. L. Mirvis P. H. (2011). A framework for the human resources role in managing culture in mergers and acquisitions. *Hum. Resour. Manage.* 50 859–877. 10.1002/hrm.20445

[B76] Maslach C. Jackson S. E. (1981). The measurement of experienced burnout. *J. Organ. Behav.* 2 99–113. 10.1002/job.4030020205

[B77] Maslach C. Leiter M. P. (2008). Early predictors of job burnout and engagement. *J. Appl. Psychol.* 93 498–512. 10.1037/0021-9010.93.3.498 18457483

[B78] Mattingly V. Kraiger K. (2019). Can emotional intelligence be trained? A meta-analytical investigation. *Hum. Resour. Manage. Rev.* 29 140–155. 10.1016/j.hrmr.2018.03.002

[B79] Mayer J. D. Salovey P. (1997). “What is emotional intelligence?” in *Emotional Development and Emotional Intelligence: Implications for Educators*, eds Salovey P. Sluyter D. (New York, NY: Basic Books), 3–31.

[B80] Mayer J. D. Caruso D. R. Salovey P. (2016). The ability model of emotional intelligence: Principles and updates. *Emot. Rev.* 8 290–300. 10.1152/physrev.00041.2012 24382883

[B81] Mérida-López S. Extremera N. (2017). Emotional intelligence and teacher burnout: A systematic review. *Int. J. Educ. Res.* 85 121–130. 10.1016/j.ijer.2017.07.006

[B82] Meyer J. P. Allen N. J. Smith C. A. (1993). Commitment to organizations and occupations: Extension and test of a three-component conceptualization. *J. Appl. Psychol.* 78 538–551. 10.1037/0021-9010.78.4.538

[B83] Miao C. Humphrey R. H. Qian S. (2017). A meta-analysis of emotional intelligence and work attitudes. *J. Occup. Organ. Psychol.* 90 177–202. 10.1111/joop.12167

[B84] Miao C. Humphrey R. H. Qian S. (2018). A cross-cultural meta-analysis of how leader emotional intelligence influences subordinate task performance and organizational citizenship behavior. *J. World Bus.* 53 463–474. 10.1016/j.jwb.2018.01.003

[B85] O’Neill O. A. Rothbard N. P. (2017). Is love all you need? The effects of emotional culture, suppression, and work–family conflict on firefighter risk-taking and health. *Acad. Manage. J.* 60 78–108. 10.5465/amj.2014.0952

[B86] O’Reilly C. A. III Chatman J. Caldwell D. F. (1991). People and organizational culture: A profile comparison approach to assessing person-organization fit. *Acad. Manage. J.* 34 487–516. 10.5465/256404

[B87] Paauwe J. (2009). HRM and performance: Achievements, methodological issues and prospects. *J. Manage. Stud.* 46 129–142. 10.1111/j.1467-6486.2008.00809.x

[B88] Pallant J. (2011). *SPSS survival manual: A step by step guide to data analysis using SPSS* 4th Edn. Maidenhead: Open University Press.

[B89] Ployhart R. E. Hale D. Jr. Campion M. C. (2014). “Staffing within the social context,” in *The Oxford handbook of organizational climate and culture*, eds Schneider B. Barbera K. M. (Oxford Oxford University Press), 23–43.

[B90] Podsakoff P. M. MacKenzie S. B. Podsakoff N. P. (2012). Sources of method bias in social science research and recommendations on how to control it. *Annu. Rev. Psychol.* 63 539–569. 10.1146/annurev-psych-120710-100452 21838546

[B91] Podsakoff P. M. MacKenzie S. B. Lee J. Y. Podsakoff N. P. (2003). Common method biases in behavioral research: A critical review of the literature and recommended remedies. *J. Appl. Psychol.* 88 879–903. 10.1037/0021-9010.88.5.879 14516251

[B92] Quinn R. E. Rohrbaugh J. (1983). A spatial model of effectiveness criteria: Towards a competing values approach to organizational analysis. *Manage. Sci.* 29 363–377.

[B93] Rich B. L. Lepine J. A. Crawford E. R. (2010). Job engagement: Antecedents and effects on job performance. *Acad. Manage. J.* 53 617–635. 10.5465/AMJ.2010.51468988

[B94] Roccas S. Sagiv L. (eds) (2017). *Values and behavior: Taking a cross cultural perspective.* Berlin: Springer. 10.1007/978-3-319-56352-7

[B95] Roulin N. Krings F. (2020). Faking to fit in: Applicants’ response strategies to match organizational culture. *J. Appl. Psychol.* 105 130–145. 10.1037/apl0000431 31233316

[B96] Rousseau D. M. Greller M. M. (1994). Human resource practices: Administrative contract makers. *Hum. Resour. Manage.* 33 385–401. 10.1002/hrm.3930330308

[B97] Saeed B. B. Afsar B. Hafeez S. Khan I. Tahir M. Afridi M. A. (2019). Promoting employee’s proenvironmental behavior through green human resource management practices. *Corp. Soc. Responsib. Environ. Manage.* 26 424–438. 10.1002/csr.1694

[B98] Salanova M. Bakker A. B. Llorens S. (2006). Flow at work: Evidence for an upward spiral of personal and organizational resources. *J. Happiness Stud.* 7 1–22. 10.1007/s10902-005-8854-8

[B99] Schaufeli W. (2021). The burnout enigma solved? *Scand. J. Work Environ. Health* 47 169–170. 10.5271/sjweh.3950 33604675 PMC8126437

[B100] Schaufeli W. B. Taris T. W. (2005). The conceptualization and measurement of burnout: Common ground and worlds apart. *Work Stress* 19 256–262. 10.1080/02678370500385913

[B101] Schaufeli W. B. Bakker A. B. (2004). Job demands, job resources, and their relationship with burnout and engagement: A multi-sample study. *J. Organ. Behav.* 25 293–315. 10.1002/job.248

[B102] Schaufeli W. B. Salanova M. González-Romá V. Bakker A. B. (2002). The measurement of engagement and burnout: A two sample confirmatory factor analytic approach. *J. Happiness Stud.* 3 71–92. 10.1023/A:1015630930326

[B103] Shirom A. Melamed S. (2006). A comparison of the construct validity of two burnout measures in two groups of professionals. *Int. J. Stress Manage.* 13 176–200. 10.1037/1072-5245.13.2.176

[B104] Steiger J. H. (2007). Understanding the limitations of global fit assessment in structural equation modeling. *Pers. Individ. Dif.* 42 893–898. 10.1016/j.paid.2006.09.017

[B105] Sterling A. Boxall P. (2013). Lean production, employee learning and workplace outcomes: A case analysis through the ability-motivation-opportunity framework. *Hum. Resour. Manage. J.* 23 227–240. 10.1111/1748-8583.12010

[B106] Sun L. Y. Aryee S. Law K. S. (2007). High-performance human resource practices, citizenship behavior, and organizational performance: A relational perspective. *Acad. Manage. J.* 50 558–577. 10.5465/amj.2007.25525821

[B107] Takeuchi R. Lepak D. P. Wang H. Takeuchi K. (2007). An empirical examination of the mechanisms mediating between high-performance work systems and the performance of Japanese organizations. *J. Appl. Psychol.* 92 1069–1083. 10.1037/0021-9010.92.4.1069 17638466

[B108] Toppinen-Tanner S. Kalimo R. Mutanen P. (2002). The process of burnout in white-collar and blue-collar jobs: Eight-year prospective study of exhaustion. *J. Organ. Behav.* 23 555–570. 10.1002/job.155

[B109] Trice H. M. Beyer J. M. (1984). Studying organizational cultures through rites and ceremonials. *Acad. Manage. Rev.* 9 653–669. 10.5465/amr.1984.4277391

[B110] Tsai Y. (2011). Relationship between organizational culture, leadership behavior and job satisfaction. *BMC Health Serv. Res.* 11:98. 10.1186/1472-6963-11-98 21569537 PMC3123547

[B111] Van der Heijden B. I. Peeters M. C. Le Blanc P. M. Van Breukelen J. W. M. (2018). Job characteristics and experience as predictors of occupational turnover intention and occupational turnover in the European nursing sector. *J. Vocat. Behav.* 108 108–120. 10.1016/j.jvb.2018.06.008

[B112] Van Wingerden J. Derks D. Bakker A. B. (2017). The impact of personal resources and job crafting interventions on work engagement and performance. *Hum. Resour. Manage.* 56 51–67. 10.1002/hrm.21758?

[B113] Weijters B. Baumgartner H. (2012). Misresponse to reversed and negated items in surveys: A review. *J. Mark. Res.* 49 737–747. 10.1509/jmr.11.0368 11670861

[B114] Wiener Y. Vardi Y. (1990). Relationships between organizational culture and individual motivation—a conceptual integration. *Psychol. Rep.* 67 295–306. 10.2466/pr0.1990.67.1.295

[B115] Wood S. Wall T. (2002). “Human resource management and business performance,” in *Psychology at work*, ed. Warr P. (London: Penguin press), 351–374.

[B116] Ye J. (2012). The impact of organizational values on organizational citizenship behaviors. *Public Pers. Manage.* 41 35–46. 10.1177/009102601204100504

